# The Role of Biodegradable Temporizing Matrix in Hand Surgery for Paediatric Recessive Dystrophic Epidermolysis Bullosa

**DOI:** 10.3390/jcm15031059

**Published:** 2026-01-29

**Authors:** Aikaterini Bini, Michael Ndukwe, Greta Piccioli, Christina Lipede, Andrea Jester

**Affiliations:** Hand and Upper Limb Service, Department of Paediatric Plastic, Hand & Reconstructive Surgery, Birmingham Women’s and Children’s NHS Foundation Trust, Birmingham B4 6NH, UK; mndukwe1@sheffield.ac.uk (M.N.); g.piccioli1@student.unisi.it (G.P.); christina.lipede2@nhs.net (C.L.); andrea.jester@nhs.net (A.J.)

**Keywords:** biodegradable temporizing matrix, BTM, skin substitute, webspace release, finger contractures, epidermolysis bullosa

## Abstract

**Background/Objectives:** The purpose of this study is to review the use of Biodegradable Temporizing Matrix (BTM) for releasing finger webspaces and flexion contractures in recessive dystrophic epidermolysis bullosa (RDEB) in paediatric patients and evaluate the short-term and mid-term results. **Methods:** Patients who underwent release of webspace fusion and flexion contractures followed by BTM application during the last two years (2022–2024) were included. The data collected included the patient demographics, previous surgical management, type of release, post-operative complications and final outcomes. BTM was used in four patients. **Results:** The functional outcomes were acceptable but temporary, as three cases required or are planned for re-operation due to recurrence. **Conclusion:** The surgical experience using BTM as a single-stage dressing/dermal substitute in hand surgery for paediatric RDEB has shown feasibility and safety regarding infection rates. To the best of our knowledge, this is the first reported study regarding the BTM use for releasing hands in epidermolysis bullosa.

## 1. Introduction

Epidermolysis bullosa (EB) is a group of rare congenital genetic conditions that result in painful blistering of the skin and mucous membranes, which occurs with minor trauma or friction. There are many types and subtypes of EB that need to be distinguished, as the management and prognosis of each can vary significantly. EB is caused by mutations in several genes; some types are autosomal dominant while others are autosomal recessive. The underlying mechanism is a defect in the attachment between or within the epidermis and dermis of the skin. There are four main types: EB simplex, dystrophic EB, junctional EB and Kindler syndrome [1].

Patients with recessive dystrophic EB characteristically suffer from blistering skin, ulcers and erosions in all areas subject to persistent or repeated friction, such as the hand. Hand deformities occur in most patients with dystrophic EB (DEB) and include adduction contractures of the first webspace, pseudosyndactyly and flexion contractures of the interphalangeal, metacarpophalangeal and wrist joints. All structures in the hand may be involved. The severity of the deformity worsens with age and surgical correction becomes increasingly difficult. Recurrent deformity occurs at varying times depending on the amount of after-care and rehabilitation [2].

There has been a longstanding discussion on how to best manage fused fingers and mitten hand deformities in this group of patients, as any intervention using their own skin will inevitably lead to a refusion, which in most cases is just a matter of time. Experienced hand surgeons have long advocated for a ‘less is more’ approach, trying to minimise the morbidity and effect on the patient, whilst achieving as much release as possible. In the past, blister skin grafts as well as full-thickness grafts have been widely used to release the first webspace and the fingers. The problems with any graft are the prevalent risk of infection and graft loss due to the wide colonization of the skin with Pseudomonas aeruginosa and other contaminants.

Biodegradable Temporizing Matrix (BTM) is a polymer dermal substitute made of a biodegradable polyurethane matrix foam covered with a non-biodegradable polyurethane sealing membrane to mimic the dermis and epidermis, respectively. When placed over a wound, it promotes vascularization and dermal regeneration [3]. BTM has been claimed to be less prone to infection and the possibility of achieving the goal of less morbidity with potentially improved operative outcomes has led to senior surgeons starting to use BTM instead of blister grafts or full-thickness grafts in some EB cases. Whilst larger areas of BTM need split-thickness skin graft coverage after 3 to 4 weeks, smaller areas seem to heal without secondary grafting being necessary.

The purpose of this study is to review the use of BTM for releasing the webspaces and flexion contractures of fingers in children suffering from recessive dystrophic EB, in cases where a skin graft is not surgically indicated, and evaluate the short-term and mid-term results.

## 2. Patients and Methods

The present study is a retrospective study. This study was registered with the Institution’s Research & Development office and in accordance with the UK National Research Ethics Service guidance, neither individual informed consent nor formal research ethics committee review was required as the study was undertaken by the direct clinical care team using information previously collected in the course of routine care (Local Reference: 35/BWC/LA/Bini). Details regarding personal information and identification remain anonymous and confidential. No recognizable features are included in the illustrations. Written consent was obtained from all the patients for the publication of their illustrations.

The selection criteria included paediatric patients with recessive DEB (RDEB) affecting the entire body including the hands, who underwent webspace and finger release followed by application of BTM.

The intra-operative findings included fusion between the fingers, including the first webspace and flexion contractures of the fingers, which in several cases were also fused to the palmar skin. Flexion contracture at the level of wrist joint was also identified in one case.

The main aim of surgery was to achieve a reasonably deep first webspace and functional position of the fingers without the need to achieve anatomical reconstruction. This approach releases the fingers to improve function, while decreasing morbidity and the burden on the patient. The first webspace was released via a horizontal incision across the webspace, with careful and meticulous release of the dermal layers. Care was taken not to breach into the fat if the decision was made to use BTM.

The interdigital webspaces were released carefully by finding the entry point, leaving the dermis intact and just separating the synechia, in most cases leading to a complete release of the webspace without blood loss.

The finger flexion contractures were released with multiple horizontal incisions down to the dermal layer along the palmar side of the fingers, until the digits were released to a functional position; avoiding though breaching into the fat, in order to protect the neurovascular bundles and keep them unexposed.

BTM was applied to all defect areas and secured with 5-0 Vicryl Rapide sutures. The tourniquet time varied between 23 and 40 min. The dressings around the fingers included silver or non-sticky dressing. Oral antibiotics were prescribed in all cases and the patients were discharged the same or the following day, if concomitant procedures, e.g., dental or gastrointestinal work, were required. The second change of dressing was performed after 5 days using the same dressing. During the third visit, the dressings were minimised. Once the silicone layer (the non-biodegradable polyurethane sealing membrane) of BTM had fallen off, standard non-sticky single-finger dressing was chosen to keep the fingers separated. Occupational therapy sessions and splinting were arranged 14 days post-operatively.

Three parameters were used to assess the treatment success: (1) maintaining thumb abduction, (2) maintaining the second-to-fourth webspaces and (3) no finger refusion/contractures. A functional assessment was performed during every session of occupational therapy. Treatment failure included thumb adduction, second-to-fourth webspace refusion, finger contractures and restriction in daily activities. If these were identified, then there was an indication for re-operation. Additionally, treatment success with BTM was generally assessed by the fact that there was no need for secondary grafting and this was the primary purpose of switching to BTM for EB hand surgery.

## 3. Results

During the study interval (2022–2024), the medical records of four consecutive patients with RDEB admitted for hand-releasing surgery were reviewed. Their demographic data were recorded. There were two male and two female patients, with an average age of 12 years (age range from 7 to 16 years old) when undergoing hand-releasing surgery with BTM application (Table 1). After the introduction of BTM in the hospital in 2022, all the RDEB patients were treated with BTM application for hand release. No grafting has been used in any RDEB patient since 2022. The total number of consecutive hands released with BTM was nine: both hands in Patient 1 (*n* = 2), both hands in Patient 2 (*n* = 2), both hands and a left-hand re-operation in Patient 3 (*n* = 3) and both hands in Patient 4 (*n* = 2).

Each patient was operated when they asked for surgery. The patients and their families were encouraged to make the decision for surgery themselves, hence the wide age variability. Re-operation was offered to the non-compliant patients, as they presented with significantly impaired hand function affecting their daily lives and they could benefit from a second surgical procedure combined with the appropriate post-operative care. Interestingly, the non-compliant group showed compliance after re-operation.

All the EB patients required ongoing follow-up due to the progression of the disease and the expected recurrence. One of them underwent re-operation and two of them are planned for re-operation in the future, while the 16-year-old patient was transferred to the adult service for continuation of care.

All the patients underwent BTM application to both hands: two of them at the same time (P1, P3) and two of them at different operative times (P2, P4). One patient required re-operation on the left hand 15 months after bilateral hand release due to recurrence (P3), while two patients are planned for re-operation in the near future (P2, P4). In past years, before the introduction of BTM, three of these patients had undergone other surgical procedures, like debridement of the fragile skin and use of blistered skin graft.

BTM incorporation required 3 to 4 weeks after its application. No evidence of infection was identified in any of these patients. Changes of dressing took place in the out-patient clinic and there was no need for general anaesthesia. All the patients were discharged at home with oral analgesia and no need for visiting the hospital for pain issues was recorded.

Patient 1 (P1) was first operated at the age of 16 years old, without having any previous hand surgery. The pre-operative findings included bilateral thumb adduction, contractures in all fingers and scars between the webspaces, excessive flexion contractures in bilateral index, middle and ring fingers and flexion contractures at both wrist joints. Bilateral hand releasing surgery and BTM application were performed, aiming for a functional outcome rather than for 100% release. Occupational therapy and splinting at night and during short periods of daytime started three weeks after the operation.

Three months post-operatively all the webspaces remained released, while the digits presented contracted and the patient was unable to wear a splint due to increased pain. The right hand presented with finger contractures, increased proximal interphalangeal (PIP) joint contractures, maintaining though the webspaces. Increased flexion in the PIP joints and wrist flexion in the right hand were also identified. Passive flexion of the right wrist to a neutral position was possible, while the digits presented with some passive stretch, limited though due to the fragility of the skin. The left hand remained with good digit extension and thumb abduction, but the metacarpophalangeal (MCP) joints and wrist joint presented with radial deviation. A paddle splint, using 3.2 mm Ezeform, was refabricated for the right hand, keeping the wrist in a neutral position and the PIP joints in as much extension as comfortable. The splint for the left hand was remoulded for a better fit, keeping the wrist in a neutral position. The splinting aimed to maintain a neutral alignment and prevent worsening of contractures. Twelve months post-operatively, the patient remained compliant with splint application and maintained good hand function bilaterally without refusion.

**Figure 1:**
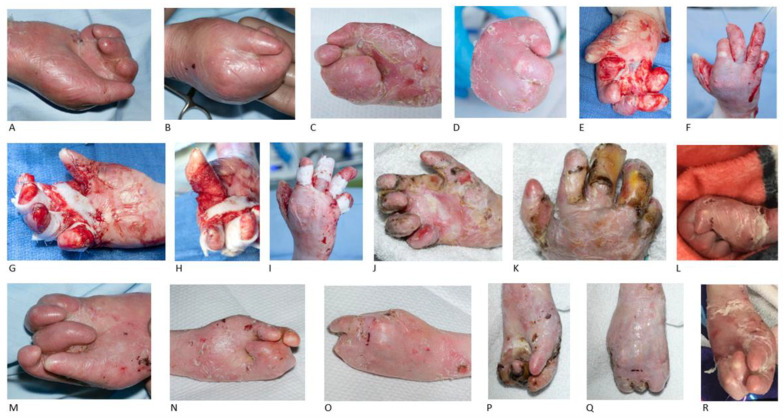
Bilateral hand release and BTM in recessive dystrophic epidermolysis bullosa. The right hand of a 9-year-old male patient with the first webspace almost completely webbed with significant finger fusions and contractures in the metacarpophalangeal, proximal and distal interphalangeal joints (**A**,**B**). The pre-operative clinical appearance of patient’s right hand at the age of 12 years old, with deterioration and progression of the disease (**C**,**D**). Intra-operative images of the first webspace and finger release of the right hand (**E**,**F**), followed by BTM application to the first webspace and circumferentially to the rest of the fingers (**G**–**I**). BTM results for the right hand 19 days (**J**,**K**) and 2 years (**L**) post-operatively, with finger contractures and refusion. The patient’s left hand at the age of 9 years old (**M**) and at the age of 12 years old (**N**,**O**), with clinical deterioration, excessive and ongoing contractures, primarily affecting the middle finger. Index finger synechia, middle, ring and little fingers significant in palm contracture and fusion, with the ring finger lying on top of little finger (**M**–**O**). Left-hand release was performed at the age of 12 years old; 2 months after the operation on the right hand. BTM results for the left hand 11 days (**P**,**Q**) and 2 years (**R**) post-operatively, with finger contractures and refusion.

Patient 2 (P2) underwent hand surgery for the first time at the age of 6 years old, where right-hand release with blister graft was performed, followed one year later by release of first webspace and all digits of left hand and application of full-thickness skin graft to the first webspace and blister graft to the remaining webspaces. The patient was re-operated at the age of 9 years old. The pre-operative findings included a contracted first webspace and pseudosyndactylised fingers flexed into the palm. Bilateral hand surgery was performed with finger syndactyly release and deepening of the first webspace, achieving separation of the thumb and fingers. The patient was not compliant with the occupational therapy and did not wear any splint. At the age of 12 years old, both hands presented fully contracted again. At that age the right hand was operated and BTM was applied, achieving partial release of the first webspace and synechia release of the second-to-fifth digits (Figure 1).

One month post-operatively the patient had a functional grasp, with a 4 cm radial abduction at the right first webspace. All the other fingers were severely contracted at the interphalangeal joints. A thermoplastic first-webspace hand-based splint from 1.6 mm Orfit was fabricated for the right hand. Splint application during the night-time and splint review every 2-3 weeks were planned.

Two months later the left hand was also surgically released with BTM use. The pre-operative findings were the following: the first webspace almost completely webbed, index finger synechia but no flexion contracture, third-to-fifth digits significant in palm contracture and fusion, with ring finger lying on top of little finger. The first, second, third and fourth digits were released without breaching into the fat, achieving an almost straight position. However, the little finger was not released, as it was buried into the palm (Figure 1).

Two weeks post-operatively the fabrication of a volar-based right-hand splint with webspaces was unachievable due to the positioning of the fingers. The patient continued with dressing between each webspace. Three months post-operatively both hands had healed. After that time the occupational therapy input was minimal, as the communication with the family was limited. Two years after surgery the patient presented with refusion and poor hand function and re-operation is planned in the near future.

**Figure 2:**
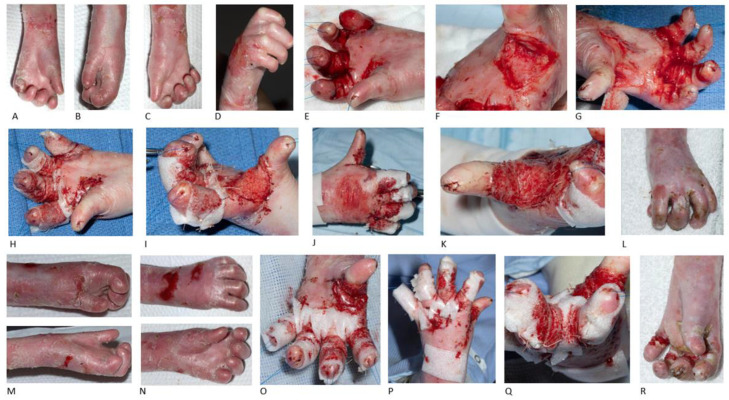
Bilateral hand release, BTM and left-hand re-operation in recessive dystrophic epidermolysis bullosa. Pre-operative clinical appearance of the left hand (**A**,**B**) and right hand (**C**,**D**) in a 6-year-old female patient with skin ulcers, tight bands, finger fusion, synechia and severe contractures in metacarpophalangeal, proximal and distal interphalangeal joints bilaterally. Intra-operative images of the first webspace and finger release in left (**E**,**F**) and right hand (**G**). BTM application to raw areas of first webspace and circumferentially to the rest of fingers of left (**H**,**I**) and right hand (**J**,**K**). Final result of the right hand 16 months after the operation (**L**). One year after the first operation, disease progression in left hand occurred, with refusion and contracture deterioration. Pre-operative (**M**,**N**) and intra-operative images after BTM application to all webspaces and circumferentially to 4 fingers of left hand (**O**–**Q**). Achievement of 80-degrees thumb abduction and significant improvement of fingers, but with remaining contractures at the proximal interphalangeal joint of around 30 degrees in all fingers except thumb (**O**). Final results of the left hand 2 months post-operatively (**R**).

Patient 3 (P3) presented initially at the age of 2 years old with very mild flexion contractures of all fingers and very mild webbing. At that point conservative management was decided, with finger wrapping individually using non-adhesive dressing and no splinting. At the age of 7 years old the patient underwent bilateral release of first webspace and fingers with BTM use. Tight bands and synechia in both hands were identified pre-operatively, while both thumbs were completely adducted (Figure 2).

One month post-operatively, the distal interphalangeal (DIP) joints were identified deviated with satisfying thumbs abduction. Good thumb opposition to the index finger with a bilateral pincer grasp, but a weak grip due to overall limited range were recorded. Night splints were fabricated with fleecy web and contour foam for the webspaces, aiming to maintain the webspaces and range of motion.

Fifteen months after the first operation, the patient remained almost 12 months without use of splint. At the age of 8 years old they had a re-operation on the left hand with release of all webspaces and palmar finger contractures with BTM application. Immediately post-operatively, an 80-degree abduction of the left thumb was achieved. The left middle finger, showing the most flexion contracture pre-operatively, had significant improvement as well. There were remaining contractures at the PIP joints of around 30 degrees in all fingers except thumb (Figure 2).

Two weeks post-operatively a thermoplastic resting splint was fabricated with the thumb slightly exposed; while strapping and fleecy web were applied to the edges. Regular follow-up was arranged every four weeks and six months after the second operation the patient remains compliant with left-hand splinting. The right hand is also planned for re-operation in the near future.

**Figure 3:**
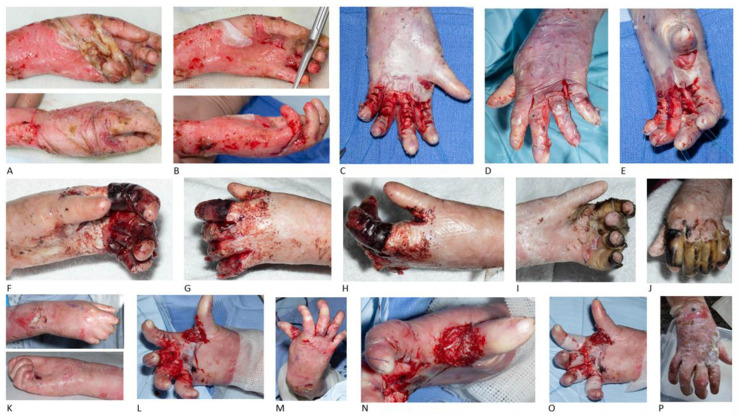
Repeated hand release and BTM in recessive dystrophic epidermolysis bullosa. The left hand of a 9-year-old female patient before (**A**) and after (**B**) debridement of fragile skin in theatre. Intra-operative images of the same patient at the age of 14 years old during sub-epidermal separation of synechia and release of second-to-fifth digits and deepening of first webspace in left hand and BTM use. The distal interphalangeal joint in the fifth digit remained slightly more flexed, while the correction of second-to-forth digits was almost straight, with a 20-degrees extension deficit in the metacarpophalangeal, proximal and distal interphalangeal joints (**C**–**E**). BTM results 4 days (**F**–**H**) and 18 days (**I**,**J**) post-operatively, with satisfying hand release. The pre-operative images of patient’s right hand at the age of 15 years old, with narrowed webspaces (**K**). First webspace and finger release of right hand with an incision in the epidermal layer and separation of pseudosyndactyly, without breaching the dermis and exposure of fat (**L**,**M**). BTM application to the first webspace (**N**) and circumferentially to the rest of the fingers, secured with 5-0 Vicryl Rapide (**O**). Final result 2 months post-operatively (**P**).

Patient 4 (P4) underwent at the age of 9 years old the first operation for left hand debridement and separation of third webspace. At the age of 14 years old, the patient presented with left-hand synechia, thumb adduction, flexion contractures in the index, middle, ring and little finger, and the fifth digit slightly more flexed. Left-hand releasing surgery with BTM application was performed, achieving correction of second-to-fourth digits, with an almost straight position and a 20-degree extension deficit. Immediately after surgery there was a remaining contracture of 20 degrees in the MCP, PIP and DIP joint levels (Figure 3).

One month post-operatively abduction was achieved, although reduced throughout the fingers in the second, third and fourth webspaces. Radial thumb abduction was also achieved, although reduced. Full extension of the PIP joints in the index, middle, ring and little fingers was recorded as well. Dressings were applied to maintain the webspaces and support finger extension, while splinting was not achievable due to the bulky dressing.

Two months post-operatively a volar-based left-hand splint with slight wrist extension, MCP joints at about 90-degrees extension and the interphalangeal joints at full extension was fabricated for application at night and during rest. Two weeks later the patient was able to pick up and stabilise objects in their hand using a disc-like grasp and was also able to use both hands together for dynamic/stable bilateral hand function tasks. A new splint was fabricated with the aim to achieve more flexion at the MCP joints. One week later, the palmar abduction of the thumb was decreased and the thumb opposition limited the ability to hold large objects. The splint was refabricated to a POSI (position of safe immobilization) splint, with the aim to get the MCP joints to 90-degrees extension.

Three months post-operatively the left hand was completely healed. There were significant limitations at the thumb webspace and flexion was limiting cylindrical and spherical grasp patterns, while the fingers were moving without fluidity. Splinting continued with application at night.

Four months post-operatively a new resting hand splint was fabricated and soft strapping was used, with the aim to maintain the available radial abduction of the thumb and the interphalangeal joint extension. Seven months post-operatively, the patient had not been wearing any splint for a couple of weeks, losing some range of motion since the last review and since surgery. Eight months post-operatively, the patient continued to be uncompliant with splinting, wishing no further occupational therapy. However, advice was given for splint application at night to maintain left-hand extension.

Fourteen months after the left-hand surgery, at the age of 15 years old, the patient underwent a right-hand release of all webspaces and BTM application. The pre-operative findings included a narrowed first webspace and all other finger webspaces. The surgery achieved an extension increase in the first webspace of more than 50 degrees, extension increase of MCP joints and PIP joints of about 40 degrees and DIP joints of 30 degrees (Figure 3).

One month post-operatively, a thermoplastic splint was fabricated for application to the right hand during the night. One week later the right splint remained fit, while a palm protector was provided for the left hand to prevent further loss of range of motion.

Two months after the second operation, the left hand presented with severe webspace contractures. A resting hand splint was fabricated for the left hand and a thermoplastic splint for the right hand. Two weeks later the left little finger was identified with flexion deterioration at the DIP joint.

Four months after the second surgery, the patient presented with difficulty in grasping and pronation/supination. The splint was adjusted with fleecy web and strapping to better support the thumb into abduction.

Five months post right-hand release, the right-hand grasp was not so wide, while the patient was able to grip with the left hand. Right-digit contractures at zone 1 of all fingers (index, middle, ring and little fingers) were identified, while contractures of left middle, ring and little fingers at zone 2 and left index finger at zone 1 were also observed. Regarding the range of motion, 45-degrees wrist extension bilaterally with full wrist flexion bilaterally and full supination/pronation bilaterally were recorded.

Seven months post right-hand release and 24 months post left-hand release, the patient continued with a resting splint for the right hand, with the plan for left-hand re-operation in the near future, due to palm and finger flexion contractures.

**Table 1:** Recessive dystrophic epidermolysis bullosa. Patients’ genetics, operations and outcomes.

Patient	Gender	Genetics/Type	Previous Operations	Hand Release and BTM	OT Results/Outcomes
P1	male	COL7A1 mutation/generalised severe	No	Bilateral hand release (16 y.o.)	12 months post-op: -Compliance with splinting -Bilateral maintaining thumb abduction -Bilateral maintaining webspaces -Bilateral digit contractures
				No further surgery	
P2 (Figure 1)	male	COL7A1 mutation/generalised severe	Right-hand release and blister graft (6 y.o.)	Right-hand release (12 y.o.)	24 months post-op: -No compliance with splinting or OT -Bilateral thumb adduction -Bilateral finger refusion -Bilateral finger severe contractures
			Left-hand release and FTSG to first webspace and blister graft to remaining webspaces (7 y.o.)	Left-hand release 2 months later (12 y.o.)	
			Bilateral hand re-operation (9 y.o.)	Re-operation planned in the near future	
P3 (Figure 2)	female	COL7A1 mutation/generalised severe	No	Bilateral hand release (7 y.o.)	12 months post 1^st^ surgery: -No compliance with splinting -Bilateral webspace refusion and finger contractures
				Left-hand re-operation (8 y.o.)	6 months post 2^nd^ surgery: -Compliance with splinting -Left thumb abduction -Maintaining left webspaces -Remaining left finger contractures
				Right hand planned for re-operation	
P4 (Figure 3)	female	COL7A1 mutation/generalised intermediate	Left-hand debridement and separation of 3rd webspace (9 y.o.)	Left-hand release (14 y.o.)	7 months post 1^st^ surgery: -No compliance with splinting -Left first webspace and thumb abduction decrease -Left finger webspaces contractures -Left finger flexion contractures
				Right-hand release 14 months later (15 y.o.)	7 months post 2^nd^ surgery: -Compliance with splinting -Maintaining right thumb abduction -Maintaining right finger webspaces -Right digit contractures
				Left hand planned for re-operation	

## 4. Discussion

This study evaluated the use of BTM for releasing finger fusion and contractures in four patients with epidermolysis bullosa. The indications, the surgical techniques used, the outcomes and the complications associated with this disease were carefully analysed to provide a comprehensive understanding of the optimal management strategies. This study included four cases of recessive dystrophic EB, all of them caused by COL7A1 mutations.

Accurate estimation of incidence and prevalence of this rare group of genetic diseases is often difficult to obtain [4,5,6]. All types of EB affect the hands; however, RDEB is the type that most commonly requires surgical intervention [7]. The hands are especially susceptible to injury in RDEB due to the shearing forces from daily activities [7]. This leads to repeated cycles of blistering, ulceration and scarring, which over time leads to a significant decrease in hand function, due to fusion of the digits, flexion contractures and loss of digits [8,9]. Deformities can occur at the skin, bone and soft-tissue level, potentially affecting all hand structures [7,8]. Repeated fibrosis and adhesions can lead to deformities, including digit pseudosyndactylies, adduction contractures in the thumb and flexion contractures of the interphalangeal, metacarpophalangeal and wrist joints [2]. Less frequently observed are extension contractures of the MCP joints, as a result of dorsal scarring [2]. Eventually, a ‘mitten’ deformity develops where the hand is encased within an epidermal cocoon [7].

The current treatment landscape for RDEB in hands ranges from surgical procedures to topical gene therapy [10]. Surgical management aims not only to counter the progression of adhesions, scarring and contractures, but also to restore normal hand anatomy and function [7,10]. Release of the thumb adduction contracture specifically offers a significant improvement for hand functionality [8]. Unfortunately, even with surgical intervention, recurrence is inevitable, which can be frustrating for the patients and their families, as well as for the health care professionals [7,8].

A common issue in RDEB is that hand surgery usually needs to be repeated due to disease recurrence. Rehabilitation must try to prevent recurrence and minimise re-operations and the surgical impact on patients’ life. Obtaining a functional thumb-index pinch is the main goal of surgical treatment [8]. The time interval for the recurrence of the thumb and finger contractures at the level of first webspace and finger webspaces has been reported to be between 12 and 24 months [7,9], while there are studies with more encouraging results, demonstrating maintenance of hand function greater than 3 years [7,8].

Once incisions are performed, a range of techniques are available for skin defect coverage. Rarely do surgeons use split-thickness skin grafts (SSGs) due to the difficulty in harvesting as well as problems during healing. More commonly, blister graft-type skin in combination with full-thickness skin grafts (FTSGs) are utilised [7]. FTSGs have been the gold standard for reducing the recurrence of contractures [7] and they are specifically recommended to be applied at the level of the first commissure [8]. However, their use is limited to small defect areas. Skin in EB patients is blistered and fragile, making autologous tissue use problematic.

More recent studies have described the use of dermal substitutes in conjunction with skin grafts, such as Matriderm and Integra [8]. These substitutes have been used under skin grafts to cover the digits and remaining commissures [8]. They are also used as an alternative to FTSGs in adults, for the first webspace and areas of exposed tendons or neurovascular bundles, in order to improve the graft-take and delay the recurrence of contractures [7]. The use of a soft silicone-coated Mepitel dressing instead of skin grafts has also been described.

Studies using dermal substitutes to treat RDEB in the hands are limited. The relevant studies most commonly describe the use of acellular dermal matrix (ADM), such as Matriderm and Integra, as skin substitutes. However, bacterial colonisation still remains the most commonly reported complication of ADMs. These materials are prone to infections, which are prevalent in EB patients [11].

Novosorb BTM is a new synthetic skin substitute that has shown promise in treating complex injuries. It is a synthetic bilayer composed of a biodegradable polyurethane open-cell foam bonded to a non-biodegradable polyurethane sealing membrane, mimicking the dermis and epidermis, respectively [12,13]. The open-cell foam acts as a scaffold allowing for vascular ingrowth [14], whilst the sealing membrane provides a mechanical and physiological barrier and allows wound drainage through small fenestrations [12,15]. It is typically applied in a two-stage process, with BTM first applied to the debrided wound bed, and then the sealing membrane is delaminated and covered with a split-thickness skin graft few weeks later [12,14]. BTM has proven to be a versatile tool with applications in extremity avulsion injuries, burns, necrotising fasciitis, tumur excision, scar revision and acute wounds [12,13,14,15,16,17].

Our experience in children has been that small areas covered with BTM healed with excellent scar formation without the need for a second procedure for grafting. The idea therefore arose to use BTM as a skin substitute without secondary grafting in EB. Initial trials included the use of BTM in small areas after release of flexion contractures of the fingers and pseudosyndactylies between the fingers, which were released without breaching the dermal layer. After initial encouraging results for replacing blister skin on the fingers, we started to use BTM for the release of the first webspace (*n* = 4) instead of full-thickness grafts. In the present study none of the patients underwent a second stage of BTM grafting with autologous skin. The results of this study show that BTM can be used more than once in case of recurrence of epidermolysis bullosa (P3). The time interval for BTM re-application depends on how rapidly the disease progresses and causes finger refusion. Dressings can keep the webspaces separated and can prolong the surgical outcome. A regular change of dressing is required every 5 to 7 days. The use of silver dressing is preferable, as it can stay longer and prevent infection.

The infection risk is lower when using BTM and the possibility for wound hypertrophy is reduced [18]. Other beneficial parameters of BTM use include successful single-stage healing, requiring 2 to 3 months for full epithelialization, without secondary grafting and donor-site morbidity. Based on these advantages and despite the high cost, which represents a significant drawback, BTM is considered an effective product, as it reduces the hospital stay, the revisionary surgery rates and shortens the median post-operative follow-up time [18]. These advantages obviously feature in patients with a healthy skin morphology or scarred tissue, but maybe not in RDEB. In this specific patient subgroup, focus should be given to higher infection rates due to wide colonization of the skin with Pseudomonas aeruginosa and other contaminants, the burden of prolonged need for dressings and regular changes, the requirement for general anaesthesia for dressing changes, the increased need of analgesia for pain control, as well as complications including hematoma, hypertrophic scarring, maceration or BTM loss.

Due to the absence of any definitive cure, the surgical managements for EB are focused on preventing complications, improving function with as little morbidity as possible and enhancing the quality of life for patients [19]. Specifically in the hands, surgery is often used to release interphalangeal adhesions, scarring and pseudosyndactyly [10]. Before BTM introduction, epidermal release (debridement) was the method of choice for many years. However, the evidence for its efficacy is not conclusive [10]. More recent studies favour preserving the innate epidermal tissue because this limits the surface area that requires healing after the surgery [10]. Some paediatric hand surgeons release only the first webspace, as this provides a stable and independent thumb, which is sufficient for many basic functions [7]. On the other hand, thumb release in not considered to be adequate in adults, where a whole-hand release is more commonly performed [7].

Kirschner wires (K-wires) and pins can also be used to straighten digits and hold favourable positions for a short period, but they can lead to complications and are not conclusively able to improve the mid-term and long-term function [7].

Dressings are integral in ensuring that the skin graft or substitute is held in place and maintaining the position of digits while protecting them [7]. This is especially important in EB to prevent shearing and damage to the skin [7]. Non-adhesive dressings are commonly used in conjunction with an antibacterial silver dressing [7]. The time until the first dressing change is approximately one week and this can be performed in the operating theatre, on the ward or in the out-patient clinic depending on the patient’s situation [10].

Most data indicates that surgery can improve or preserve hand function in RDEB. However, the evidence is not clear due to the nature of the currently available studies [7]. Generally, contracture release surgeries lead to an improvement in the grasp or pinch or restoration of prehension. Additionally, if successful, surgery offers the potential for independent finger motion and an improvement in the aesthetic appearance of the hand [7]. Hand contracture surgical release is more favourable in children, who tend to have better results than adults and adolescents, who may have joint deformities that are difficult to correct completely [7].

It is difficult to make definitive conclusions about specific surgical procedures due to the lack of literature, short follow-up periods and the rarity of the condition [20]. Nevertheless, studies have suggested that FTSGs are associated with better graft take, skin quality and recurrence rates compared to SSGs [8,20]. In a study where FTSGs were used at the level of the first commissure and palm of the hand along with SSGs in conjunction with dermal substitutes to cover the remaining commissures, digits and the remainder of the hand, maintenance of function was greater than 3 years in 57% of cases and greater than 5 years in 33% of cases [8].

The coordination of surgery and post-operative care is vital in helping reduce the time to recurrence [7]. Surgery aims to facilitate splinting, which can be used to maintain range of movement and delay deformity [7]. There are various examples of splints that can be used for this purpose, including putty, thermoplastic and dynamic splints, each with different recommendations for application [7]. Regular reviewing of splints is essential to ensure that they are applied correctly and at the indicated times to prevent injury [7]. The paediatric population may not be compliant with the necessity of continuous splinting after BTM application, which may lead to recurrence and refusion earlier than expected. Therefore, RDEB patients should be selected carefully for BTM and be aware in advance of the exact post-operative care and splinting required.

Different methods can be used to measure the outcomes of EB patients after surgery. Therefore, drawing conclusions about the different treatment options is often difficult as the outcomes are reported inconsistently [7]. Despite this, surgery can significantly improve the quality of life of these patients [20]. Measuring the time till recurrence is one method to assess the effectiveness of surgical interventions [7,8,20]. However, obtaining consistent follow-ups across multiple EB patients can be difficult due to the varied progression of the clinical manifestations [10].

Patient-Reported Outcome Measures (PROMs) only make sense if there are pre-operative and post-operative measures that show the difference. Specifically in EB it might mean only a cross-sectional point, so PROMs are not really useful for proving the benefit of the operation, since it is expected that the hand will get worse again. This is the difficulty and challenge in EB. Therefore, less burden regarding measures or scores should be put on paediatric patients, with acceptable results in a condition that will inevitably recur.

The present study has a limitation. This is a retrospective study with a small patient number with no comparison group. In the future, studies including larger cases series need to be conducted, so that we will be able to comment on the BTM results when releasing the first webspace, the second-to-fourth webspaces and the flexion contractures, and demonstrate whether the recurrence rate is lower or not when using BTM in hand surgery for RDEB.

## 5. Conclusions

This study presented preliminary results of the use of BTM as a single-stage skin substitute after release of webspaces and flexion contracture of the fingers. Whilst it might yield promising results, the need for rehabilitation and splinting is no less than with any conventional method. To the best of our knowledge, this is the first ever reported study in the literature demonstrating the role of BTM in releasing hands in paediatric patients with recessive dystrophic epidermolysis bullosa.

## Data Availability

The data presented in this study are available on request from the corresponding author.
